# In a prospective population-based study, the degree of mobility impairment during hospitalisation is associated with higher degrees of frailty

**DOI:** 10.1007/s40520-025-03178-2

**Published:** 2025-10-24

**Authors:** Samuel D. Searle, Alex Tsui, Natalie Yeo, Petronella Chitalu, Hugh Logan Ellis, Mark Rawle, Anna Seeley, Kenneth Rockwood, Daniel Davis

**Affiliations:** 1 Department of Medicine, Dalhousie University, Halifax, Canada; 2 Institute of Health Informatics, University College London, London, UK; 3 St Pancras Rehabilitation Unit, Central and North West London NHS Foundation Trust, London, UK; 4 Department of Biostatistics & Health Informatics, King’s College London, London, UK; 5 Academic Centre for Healthy Ageing (ACHA), Whipps Cross University Hospital, Barts Health NHS Trust, London, UK; 6 Nuffield Department of Primary Care Health Sciences, University of Oxford, Oxford, UK

**Keywords:** Hospital, Frailty, Mobility

## Abstract

**Background:**

Hospitals pose a high risk for frailty to develop or accelerate. Still, few community-based cohort studies follow patients before, during, and after hospitalisation. We investigated the degree of immobility during hospitalisation and its impact on subsequent frailty.

**Methods:**

In a prospective population-based cohort of individuals aged ≥ 70 from a London UK borough, we performed comprehensive community assessments at baseline and after two years. At each hospitalisation, we measured daily mobility and other clinical variables. Acute immobility burden, a summative level of poor mobility for all hospitalisations, was calculated for each participant and operationalized as low/high based on the population median. A frailty index was calculated for all participants during baseline and follow-up assessments. We estimated the effect of these exposures on follow-up frailty index scores using linear regression.

**Results:**

We included 1177 participants. Those admitted (*N* = 114) were assessed over 1999 bed-days. The degree of baseline frailty had the largest association with subsequent frailty. However, a high immobility burden during hospitalisation was consistently related to additional increases in frailty (low burden: β = 0.02 per unit increase in FI (95%CI: -0.002-0.04), high burden: β = 0.07, (95%CI: 0.041-0.10)). Immobility burden remained associated with subsequent frailty even when limiting the analysis to: those who were independently mobile; the first seven days of hospitalisation; and accounting for illness severity. High immobility burden was prognostic of subsequent death.

**Conclusions:**

The degree of immobility during hospitalisation, a potentially modifiable risk factor, may determine whether hospitalisation contributes to increasing frailty.

**Supplementary Information:**

The online version contains supplementary material available at 10.1007/s40520-025-03178-2.

## Introduction

Frailty is crucial to understanding our ageing population because it identifies individuals at risk for a broad range of adverse outcomes [1]. Frailty transition occurring though acute illness is not often quantified, and very few studies have prospectively incorporated community and hospitalisation information.

In community-dwelling samples, frailty typically develops over years and is associated with several factors (e.g., age, sex, comorbidities, cognitive function) [2, 3]. In this setting, hospitalisation appears strongly associated with worsening frailty. In hospital cohort studies, frailty is prevalent in several populations [4–6]. Individuals who previously were fit have been found to have a notable burden of frailty following hospitalisation. At its inception, the DELPHC study was unique in being a population-based cohort that followed people through each day of hospitalisation and ascertained relevant health information on individuals living with various degrees of fitness/frailty. Because hospitalisation is a potent risk factor for frailty, we considered what types of hospitalisations were associated with developing or accelerating frailty. Higher acuity hospitalisations present a potential risk from both a theoretical and observational perspective. For instance, in patients admitted to critical care units, frailty is an essential marker for adverse outcomes, and the development of frailty is common in previously robust patients [7]. 

Although older patients often present acutely with reduced mobility or delirium, rather than dyspnoea, fever or pain, these core presentations do not generally feature in our reification of acute illness [8, 9]. The degree to which inpatients are impaired when they move, whether independently or through assessment, is associated with adverse outcomes [10–12]. Older inpatients with deteriorating mobility on the first days of admission for acute illness have an expected mortality of 70% at one-month [13]. Further, the degree of immobility can indicate delirium in patients with dementia [14] and is a marker of delirium severity [15]. Increased hospital mobilisation is linked to shorter lengths of stay and functional independence [16, 17]. 

Although mobility is a distinct and measurable entity, cross-sectionally, mobility is a significant component of frailty when patients are clinically stable. During acute illness, however, mobility, as a measure of function, appears to depend on both baseline frailty status and the nature and severity of acute illness [18, 19]. Here, we investigate over two years whether cumulative immobility (immobility burden) during hospitalisation is associated with subsequent frailty in order to distinguish between ‘at-risk’ and ‘safe’ hospital admissions on a community population level. We hypothesized that the burden of hospitalised immobility would demarcate at-risk admissions for subsequent increased frailty.

## Methods

### Study design and participants

The Delirium and Population Health Informatics Cohort (DELPHIC) study [20, 21] is a prospective population-based sample initiated in March 2017 in the borough of Camden (London, UK). Eligible participants were Camden residents aged ≥ 70 years. Participants were excluded if they had severe hearing impairment or aphasia, were in the terminal phase of illness (expected life expectancy of < 6 months), or could not speak English sufficiently well to undertake a cognitive assessment. Participants were primarily enrolled via general practitioner lists (80%) or, to include a greater range of cognitive impairment and frailty, from memory clinics (10%) and recent hospital discharges (10%). DELPHIC’s primary outcome was detecting a meaningful change in cognitive testing at a two-year follow-up. The protocol received approval from an NHS Research Ethics Committee (16/LO/1217) and the Health Research Authority (IRAS 164446).

Baseline health assessments were performed in the community by telephone or home visit, with identical follow-up two years later. Participants admitted to hospital were automatically flagged to be seen daily (excluding public holidays and weekends) by a trained clinical researcher. Several health variables were assessed daily in hospital, including mobility, cognition, and physiological measures. Individuals, or their nominated proxies, gave consent or agreement to participate. Death notification was from the NHS Spine, a statutory register for all deaths in England, and were cross-referenced with local hospital electronic records systems (last update 21st June 2021).

### Measures

Frailty was measured at baseline and follow-up using a frailty index (FI) [21, 22] (Supplementary Table 1). This previously published FI, created using a standard process to ensure validity [23]was modified to exclude mobility deficits. We did this to avoid collinearity with immobility burden, a relevant exposure in this study. The same items in both the baseline and follow-up frailty indices were used.

Mobility was assessed using the Hierarchical Assessment of Balance and Mobility (HABAM). The HABAM measures the highest daily attained performance in balance (21 points), transfers (18 points), and mobility (28 points), operating as an integrated measure of mobility. The HABAM was ascertained prospectively daily during hospitalisation. The mobility measured is functional, not intensity-based. Immobility was defined using the inverse of the HABAM, where higher scores indicate poorer mobility [24, 25]. 

We used the National Early Warning Score (NEWS, version 1) which is a composite scoring system that uses physiological measurements to assess and monitor acute illness severity in hospitalised patients [26]. NEWS, a standard assessment tool mandated to be used at least daily by the NHS for all acute inpatient units, adds clinically abnormal indices (heart rate, blood pressure, respiratory rate, oxygen saturation, supplemental oxygen requirements, alertness), giving a score from 0 to 20. The index of multiple deprivation (2019) is an ecological measure of overall deprivation using 37 separate indicators across seven domains (income, employment, health, crime, education, barriers to services, and living environment). It is used throughout England and represents population sizes between 1200 and 3000 people [27]. 

### Statistical analysis

#### Outcome measure

Frailty A frailty index was used to quantify baseline (exposure) and follow-up (outcome). Assuming an alpha of 0.05, a sample size of at least 82 hospitalisations would be required to detect the effect of immobility on mortality with a power of 80%. [28].

## Exposures

Immobility burden We quantified the total immobility burden during hospitalisation, measuring duration and severity, by summing daily immobility scores across inpatient assessments. This was additive across multiple admissions to include all immobility in the hospital during the two years of study enrollment. No mobility measurements were included following any transfer to subacute care units. No metrics were available on days individuals were waiting before transfer to subacute care.

Notionally, a low or high immobility burden could differentiate the type of hospital admission. As the HABAM had no established method to measure cumulative mobility burden during hospitalisation, we implemented the metric as follows: participants who were not hospitalised had no hospital immobility burden. Otherwise, for each participant, we calculated immobility burden by summing their daily HABAM scores across all hospital admissions. Hospitalised immobility burden was then dichotomised as high or low, based on the median score.

### Missing data

In keeping with previous analyses, missing hospitalisation data during weekends and public holidays were assumed to be missing at random. Mobility data were forward and backfilled for weekends (Friday carried over to Saturday and data on Sunday from Monday) and public holidays for up to four days [21]. 

### Models

Frailty: We used linear regression to estimate the frailty index after two years in the study, adjusted by age, sex and baseline frailty). The main exposure was hospitalised immobility burden level (none/low/high) during the course of the 2-year study. Sensitivity analysis included excluding any participants with elective admissions, using immobility burden (continuous), mobility burden limited by the first 7 days of hospitalisation, correcting for baseline mobility status (mobility component of the Barthel Index), index of multiple deprivation and National Early Warning Score and only including those who were independently mobile at baseline assessment.

Additional analysis: Differences in baseline and follow-up mobility are reported to check for meaningful changes in mobility throughout the study that might be explained by immobility while not hospitalised.

We used R (4.0.2), Python (3.7.6) and Stata (17.0) for all analyses.

## Results

**Fig. 1:**
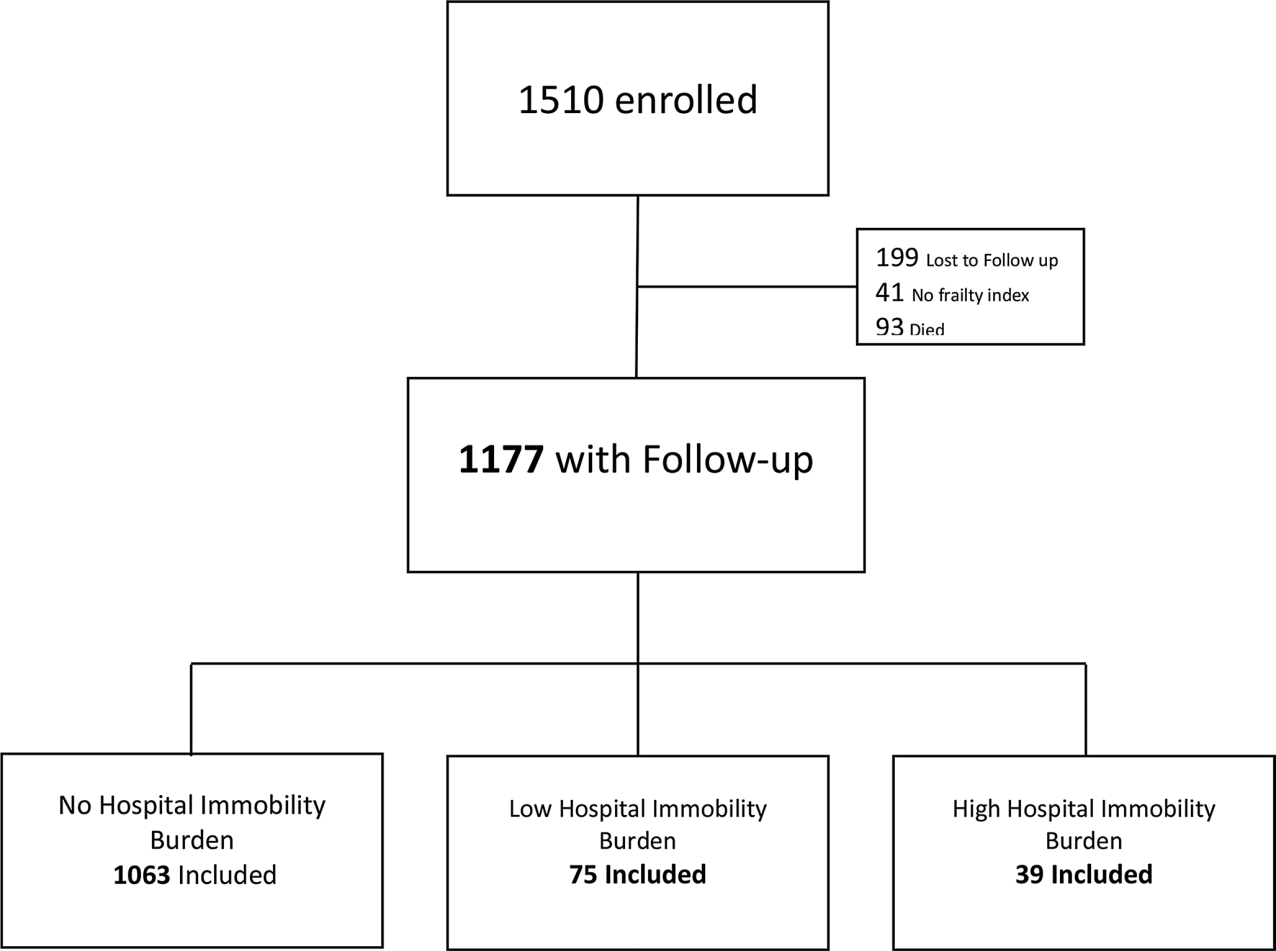
Cohort derivation from the delirium and population health informatics cohort (DELPHIC)

A total of 1177 participants were included in this analysis. Ninety-three participants of the original cohort study died before their 2-year follow-up; others were lost to attrition (*n* = 199) (Fig. 1). Higher immobility burden had higher study mortalty (53.6%, 19.4% and 2.7% for high, low and no hospital immobility burden respectively). Those not included in the final analysis were older, frailer at baseline, and had more limitations in their activities of daily living and mobility dependence at enrollment. Most people remained in the community without incident hospitalisation during the study period (*n* = 1063). The 114 hospitalised participants experienced 167 hospitalisations. Admitted participants tended to be older and frailer (Table 1). During the study period, there were 1,999 person-days of inpatient assessment.

**Table 1:** Baseline characteristics of the study population

	Overall	Not Hospitalised	Low Hospital Immobility	High Hospital Immobility
n	1177	1063	75	39
Age, mean (SD)	78.1 (5.7)	77.9 (5.6)	78.7 (5.6)	82.8 (6.3)
Female, n (%)	682 (57.9)	617 (58.0)	42 (56.0)	23 (59.0)
Barthel index, median [Q1,Q3]	17.0 [17.0,17.0]	17.0 [17.0,17.0]	17.0 [17.0,17.0]	17.0 [15.5,17.0]
Index of Multiple Deprevation, n (%)				
First decile	249 (21.4)	232 (22.1)	11 (14.7)	6 (15.4)
Second decile	252 (21.6)	228 (21.7)	18 (24.0)	6 (15.4)
Third decile	241 (20.7)	223 (21.2)	10 (13.3)	8 (20.5)
Fourth decile	203 (17.4)	176 (16.8)	17 (22.7)	10 (25.6)
Fifth decile	219 (18.8)	191 (18.2)	19 (25.3)	9 (23.1)
Frailty Index, median [Q1,Q3]	0.12 [0.06,0.21]	0.12 [0.06,0.19]	0.19 [0.12,0.28]	0.28 [0.19,0.41]
Baseline Mobility (Barthel), median [Q1,Q3]	3.0 [3.0,3.0]	3.0 [3.0,3.0]	3.0 [3.0,3.0]	3.0 [3.0,3.0]
Mean NEWS Score, median [Q1,Q3]	N/A	N/A	0.5 [0.0,1.5]	0.9 [0.5,1.5]

In those admitted to hospital, the immobility score had a median of 50 (IQR 40 to 56) out of 67 possible points. This would be clinically equivalent to a participant being able to sit up in bed independently, maintain this statically and require one person to assist with transfers out of bed. The median hospitalised immobility burden during the 2-year study duration was 145. This would be the equivalent of a participant being a full lift and unable to position themselves in bed for just over two days of hospitalisation in the intervening two years of the study, but less in-hospital immobility if spread over more days, acutely ill in hospital. The proportion of the population who were independently mobile during the baseline and two-year follow-up assessment was 99% and 96%, 99% and 93%, and 92% and 79% in the nonhospitalised, admissions with low burden of hospital immobility, and admissions with high burden of hospital immobility groups, respectively.

**Fig. 2:**
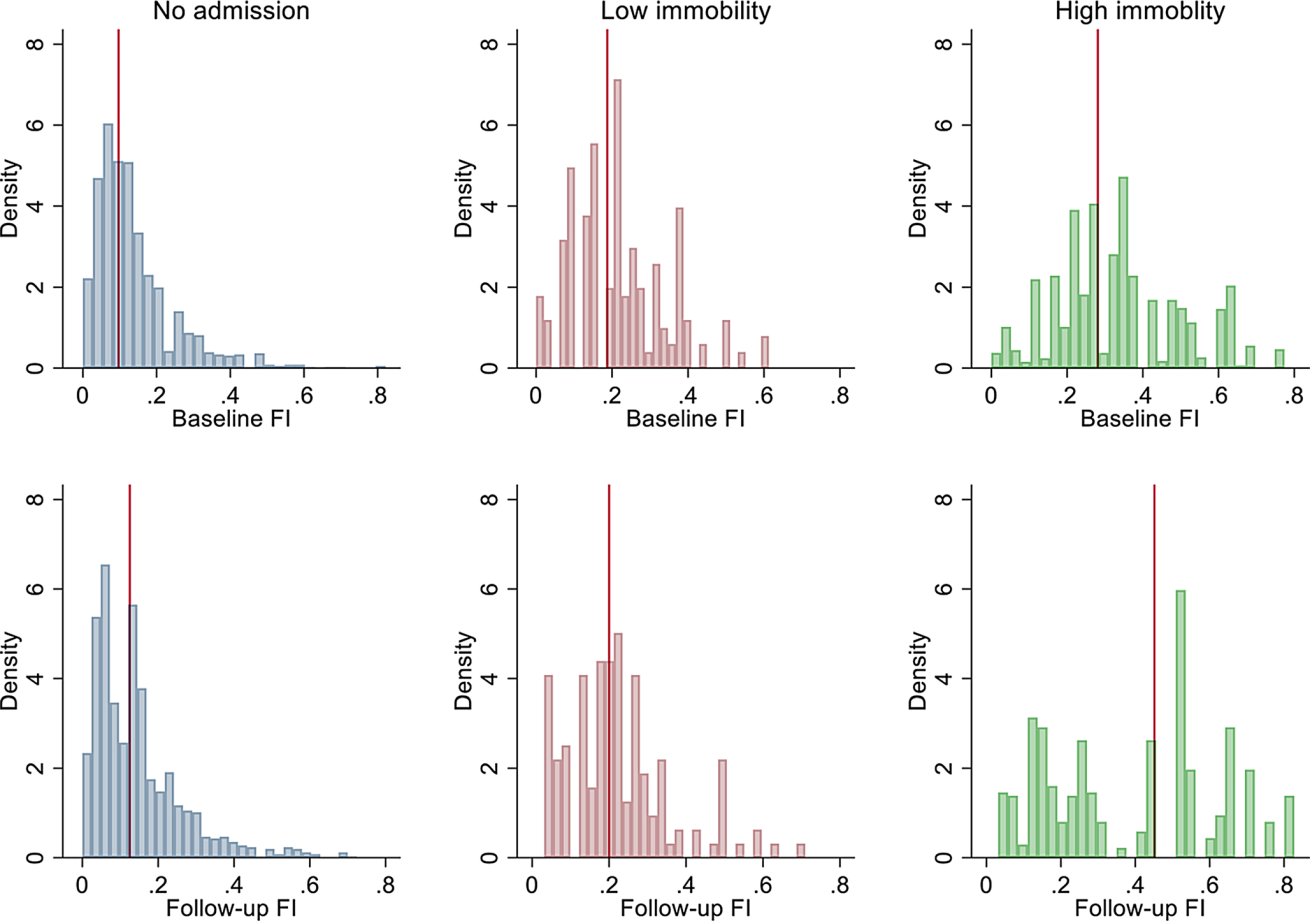
Frailty Index distributions at baseline and follow-up, by degree of inpatient immobility. The median frailty level significantly shifts to the right (more frail) in the case of a high level of immobility burden during hospitalisation. Low hospital immobility burden appears to not lead to an increasing burden of frailty over a 2 year period

For the level of frailty at follow-up, high immobility burden was associated with worse FI scores (low burden: β = 0.018, 95%CI: -0.002-0.038 and high burden: β = 0.069, 95%CI: 0.041–0.097) (Table 2). Interpreted, a high immobility burden would contribute an additional 0.07 to the frailty index score or 2.24 deficts to the deficit burden. In our model, hospitalisations with high immobility burdens have the same effect size regarding frailty progression as does an additional 20 years of ageing. This was consistent across sensitivity analyses – when accounting for baseline mobility, removing individuals who were not independently mobile at baseline, removing individuals who had any elective admissions, only for immobility measured in the first 7 days of hospital admission, and the index of multiple deprivations (Supplemental Table 2). Likewise, mean NEWS, being admitted (the combined individuals with low and high immobility) was also associated with subsequent frailty. After adjusting for immobility and NEWS, only a high level of immobility during hospitalisation appeared to be related to subsequent frailty. The two-year increase in FI for the non-hospitalised sample was 0.02, whereas the average increase in those with high immobility was 0.07 (Fig. 2).

**Table 2:** Linear regression for follow-up frailty. Baseline frailty index for the purpose of the analysis was multiplied by 10. Participants admitted were dichotomized into low and high at the median hospitalised immobility burden score of 145. Age was normalized from the minimum enrollment age (70 years old). NEWS is the National early warning score version 1 mean for all inpatient days

Model 1				
	Coefficient	95% CI		P Value
Frailty Index (per 10% increase)	0.072	0.067	0.077	< 0.001
Age (per year)	0.003	0.002	0.004	< 0.001
Women (reference to men)	-0.009	-0.019	0.001	0.064
No Hospitalised Immobility	[ref]	-	-	-
Low Hospitalised Immobility	0.018	-0.002	0.038	0.083
High Hospitalised immobility	0.069	0.041	0.097	< 0.001
Model 2				
	Coefficient	95% CI		P Value
Frailty Index (per 10% increase)	0.072	0.067	0.077	< 0.001
Age (per year)	0.003	0.002	0.004	< 0.001
Women (reference to men)	-0.010	-0.019	0	0.053
No Hospitalised Immobility	[ref]	-	-	-
Low Hospitalised Immobility	0.007	-0.018	0.031	0.584
High Hospitalised immobility	0.057	0.026	0.089	< 0.001
NEWS score	0.011	-0.003	0.026	0.131

## Discussion

In this population-based prospective cohort, over two years of observation, higher immobility during hospitalisation was associated with higher degrees of frailty. This effect was not apparent in individuals with minimal hospitalised immobility. A low level of immobility was not associated with a subsequently increased frailty burden, potentially suggesting that regardless of pre-hospitalisation frailty, not all hospital admissions in older adults increase frailty.

Despite this seemingly straightforward result, very few clinical acute care inpatient environments track or trigger medical concerns when mobility remains low. Also, contrary to some teaching, not all hospital admissions appear harmful to frail older patients [29]. True to our understanding of frailty, frailty is associated with adverse outcomes; however, when experiencing an adverse outcome (hospitalisation), mobility may appear to track outcomes. This is also consistent with the observation that, in patients with delirium, change in mobility tracks the overall course of recovery [15]. We note too that poor hospital mobility is potentially modifiable [30–32]. 

Our analyses are subject to certain limitations. Immobility during all hospital admissions were included in the analysis of which approximately 12 appeared to be elective surgical admissions as opposed to acute medical/surgical illness. Our study focuses on change within a hospital episode as the primary setting for capturing acute illness. This potential immortality bias may have had little impact, considering the minimal change in those independently mobilizing in the non-hospitalised group during the 2-year follow-up. This representative community study is a single population group and may not be externally generalisable. Our chosen operationalised exposure has been validated (HABAM), but no current studies have operationalized hospitalised immobility burden as an integrative measure using the HABAM. Further validation will be required. We also recognise that immobility over time is less clinically translatable than a single-point measurement. Despite these, these data are the first to discriminate, in a population-based study, hospitalisation risks by immobility to subsequent frailty.

Unsurprisingly, baseline frailty was the most significant risk factor for frailty in two years. Usually, deficit accumulation is somewhere in the 3–4% per year from previous studies, depending on the population [33]. Here, we identified that only high levels of in-hospital immobility resulted in an increased frailty burden. We propose three potential explanations provided our results are valid. First is that some hospitalisations are not harmful for frailty development and this is particularly marked not necessary by illness severity, but rather low levels of immobility during hospitalisation. A second possible explanation is that there is a potential dose response to immobility during hospitalisation, and simply, we have too few numbers of hospitalised patients to determine whether or not low levels of immobility lead to future frailty within two years. Another intriguing idea, which is relatively well understood in clinical experience but with relatively low data evidence, is that immobility is a marker for illness severity, much more specific to frail older individuals than traditional physiological markers of illness severity.

Potential implications of these results could be important for decision-making and care planning at the time of presentation to hospital, before presentation to hospital and during hospital stay, bearing in mind that, yes, hospitalisations can be harmful for frail older individuals with respect to mortality and frailty. Still, not all hospitalisations appear to be harmful in this way from the community-dwelling sample. Additionally, many frailer, older individuals are focused on avoiding age-related health deficits and accumulation, associated disability, as it might be more of a concern than death. As such, this would support the idea that older individuals with relatively good mobility during a hospitalisation are likely to survive and not compound frailty.

Note sure what this text pertains to: 1510 enrolled**1177** with Follow-up199 Lost to Follow up41 No frailty index93 DiedLow Hospital ImmobilityBurden**75 Included**High Hospital Immobility Burden**39 Included**No Hospital Immobility Burden**1063** Included.

## Electronic supplementary material

Below is the link to the electronic supplementary material.

Supplementary Material 1

## Data Availability

Study protocols, consent forms and case report forms are available at https://portal.dementiasplatform.uk/.
