# Supplementary material for: In a prospective population-based study, the degree of mobility impairment during hospitalisation is associated with higher degrees of frailty
Source: Aging Clin Exp Res. 2025 Oct 24;37(1):297. doi: 10.1007/s40520-025-03178-2 (PMC12552269; doi:10.1007/s40520-025-03178-2)
Supplement: Supplementary file 1 — Supplementary Material 1 [file 40520_2025_3178_MOESM1_ESM.docx]

**Supplementary Figure 1.** Immobility examples conferring high immobility burden. Any additional hospitalised immobility, either by a worse score already shown or by an additional hospitalised day with any immobility, would also be considered high immobility burden.

**Supplementary Table 1.** Prevalence of frailty index (FI) deficits at baseline and follow-up. Items removed from previously used FI were the ability to ambulate, transfer, and walk upstairs [ref].

| **Frailty index deficit** | **Baseline prevalence** | **Follow-up prevalence** |
| --- | --- | --- |
| General Health (fair/poor/very poor) | 14% | 20% |
| Myocardial infarction | 20% | 21% |
| Hypertension | 49% | 55% |
| Diabetes | 11% | 12% |
| Stroke | 8% | 11% |
| Cancer | 24% | 29% |
| COPD | 12% | 13% |
| Delirium | 10% | 12% |
| Near vision | 2% | 6% |
| Distant vision | 7% | 8% |
| Conversational hearing | 2% | 3% |
| Hearing in noisy room | 26% | 37% |
| Urinary Incontinence | 10% | 11% |
| Accidental fall | 36% | 28% |
| Weight loss | 1% | 4% |
| Feeding | 1% | 2% |
| Making hot drink | 2% | 4% |
| Washing up | 3% | 8% |
| Managing finances | 4% | 4% |
| Laundry | 6% | 12% |
| Shopping | 13% | 14% |
| Social activities | 8% | 11% |
| Gardening | 37% | 33% |
| Polypharmacy | 31% | 31% |
| Grooming | 1% | 3% |
| Bathing | 2% | 4% |
| Dressing | 2% | 3% |
| Toileting | 1% | 2% |
| Immediate Recall (<4 words) | 31% | 26% |
| Delayed Recall (<2 words) | 29% | 26% |
| Verbal fluency (<=10 animals) | 8% | 17% |
| Verbal fluency (<=10 letter words) | 17% | 22% |
| All % refer to impairments (deficits). | | |

**Supplementary Table 2. Sensitivity Analysis.** Associations between baseline immobility and follow-up Frailty Index.

|  | **β** | **95% CI** | | **p** |
| --- | --- | --- | --- | --- |
| Baseline Frailty Index | 0.071 | 0.066 | 0.076 | <0.001 |
| Age (per year) | 0.003 | 0.002 | 0.004 | <0.001 |
| Sex (women cf men) | -0.008 | -0.018 | 0.002 | 0.175 |
| No admission | [ref] |  |  | ref |
| Low immobility burden | 0.020 | -0.001 | 0.042 | 0.060 |
| High immobility burden | 0.077 | 0.047 | 0.106 | <0.001 |
| *Exlcuded if any participants if they had any elective admission. This was identified post hoc based on recorded admission diagnosis. Fourteen participants were excluded. | | | | |

|  | **β** | **95% CI** | | **p** |
| --- | --- | --- | --- | --- |
| Baseline Frailty Index | 0.081 | 0.075 | 0.086 | <0.001 |
| Baseline mobility (per level) | 0.001 | -0.023 | 0.026 | 0.909 |
| Age (per year) | 0.004 | 0.003 | 0.005 | <0.001 |
| Sex (women cf men) | -0.007 | -0.017 | 0.003 | 0.175 |
| No admission | [ref] |  |  | [ref] |
| Low immobility burden | 0.019 | -0.001 | 0.040 | 0.074 |
| High immobility burden | 0.066 | 0.038 | 0.093 | <0.001 |
| Mobility at baseline was an ordinal variable ranging from immobile, dependent on others, independent if using an aid, to independently able. | | | | |

|  | **β** | **95% CI** | | **p** |
| --- | --- | --- | --- | --- |
| Baseline Frailty Index | 0.072 | 0.075 | 0.086 | <0.001 |
| Age (per year) | 0.003 | 0.003 | 0.005 | <0.001 |
| Sex (women cf men) | -0.009 | -0.017 | 0.003 | 0.062 |
| No admission | [ref] |  |  | [ref] |
| Low immobility burden | 0.022 | 0.001 | 0.041 | 0.043 |
| High immobility burden | 0.064 | 0.036 | 0.093 | <0.001 |
| Immobility Burden Levels – only the frist 7 days of hospitalisation included in analysis | | | | |

|  | **β** | **95% CI** | | **p** |
| --- | --- | --- | --- | --- |
| Baseline Frailty Index | 0.072 | 0.067 | 0.077 | <0.001 |
| Age (per year) | 0.003 | 0.002 | 0.004 | <0.001 |
| Sex (women cf men) | -0.009 | -0.019 | 0.001 | 0.07 |
| Immobility Burden | 0.009 | 0.005 | 0.013 | <0.001 |
| Immobility Burden as a continuous variable | | | | |

|  | **β** | **95% CI** | | **p** |
| --- | --- | --- | --- | --- |
| Baseline Frailty Index | 0.072 | 0.067 | 0.077 | <0.001 |
| Age (per year) | 0.003 | 0.002 | 0.004 | <0.001 |
| Sex (women cf men) | -0.009 | -0.019 | 0.001 | 0.07 |
| Admission | 0.034 | 0.018 | 0.051 | <0.001 |
| Admission – any admission | | | | |

|  | **β** | **95% CI** | | **p** |
| --- | --- | --- | --- | --- |
| Baseline Frailty Index | 0.072 | 0.067 | 0.077 | <0.001 |
| Age (per year) | 0.003 | 0.002 | 0.004 | <0.001 |
| Sex (women cf men) | -0.010 | -0.019 | 0 | 0.053 |
| No admission | [ref] |  |  | [ref] |
| Low immobility burden | 0.007 | -0.018 | 0.031 | 0.58 |
| High immobility burden | 0.057 | 0.026 | 0.089 | <0.001 |
| NEWS | 0.011 | -0.003 | 0.026 | 0.13 |
| NEWS – National Early Warning Score mean | | | | |

|  | **β** | **95% CI** | | **p** |
| --- | --- | --- | --- | --- |
| Baseline Frailty Index | 0.071 | 0.066 | 0.076 | <0.001 |
| Age (per year) | 0.003 | 0.002 | 0.004 | <0.001 |
| Sex (women cf men) | -0.009 | -0.019 | 0.001 | 0.073 |
| No admission | [ref] |  |  | [ref] |
| Low immobility burden | 0.018 | -0.002 | 0.038 | 0.084 |
| High immobility burden | 0.069 | 0.041 | 0.097 | <0.001 |
| IMDD | 0.003 | -0.001 | 0.006 | 0.128 |
| IMDD – Index of Multiple Deprevation Quintiles | | | | |
